# Ebola VP40 in Exosomes Can Cause Immune Cell Dysfunction

**DOI:** 10.3389/fmicb.2016.01765

**Published:** 2016-11-07

**Authors:** Michelle L. Pleet, Allison Mathiesen, Catherine DeMarino, Yao A. Akpamagbo, Robert A. Barclay, Angela Schwab, Sergey Iordanskiy, Gavin C. Sampey, Benjamin Lepene, Philipp A. Ilinykh, Alexander Bukreyev, Sergei Nekhai, M. Javad Aman, Fatah Kashanchi

**Affiliations:** ^1^Laboratory of Molecular Virology, School of Systems Biology, George Mason University, Manassas, VA, United States; ^2^Department of Physiological Sciences, Eastern Virginia Medical School, Norfolk, VA, United States; ^3^Department of Microbiology, Immunology and Tropical Medicine, Research Center for Neglected Diseases of Poverty, George Washington University School of Medicine and Health Sciences, Washington, DC, United States; ^4^Department of Medicine, University of North Carolina HIV Cure Center, University of North Carolina at Chapel Hill School of Medicine, Chapel Hill, NC, United States; ^5^Ceres Nanosciences Inc., Manassas, VA, United States; ^6^Department of Pathology, University of Texas Medical Branch at Galveston, Galveston, TX, United States; ^7^Microbiology and Immunology, University of Texas Medical Branch at Galveston, Galveston, TX, United States; ^8^Galveston National Laboratory, University of Texas Medical Branch at Galveston, Galveston, TX, United States; ^9^Department of Medicine, Center for Sickle Cell Disease, Howard University, Washington, DC, United States; ^10^Integrated BioTherapeutics, Inc., Gaithersburg, MD, United States

**Keywords:** Ebola virus, EBOV, VP40, VLP, exosomes, ESCRT, microRNA, apoptosis

## Abstract

Ebola virus (EBOV) is an enveloped, ssRNA virus from the family *Filoviridae* capable of causing severe hemorrhagic fever with up to 80–90% mortality rates. The most recent outbreak of EBOV in West Africa starting in 2014 resulted in over 11,300 deaths; however, long-lasting persistence and recurrence in survivors has been documented, potentially leading to further transmission of the virus. We have previously shown that exosomes from cells infected with HIV-1, HTLV-1 and Rift Valley Fever virus are able to transfer viral proteins and non-coding RNAs to naïve recipient cells, resulting in an altered cellular activity. In the current manuscript, we examined the effect of Ebola structural proteins VP40, GP, NP and VLPs on recipient immune cells, as well as the effect of exosomes containing these proteins on naïve immune cells. We found that VP40-transfected cells packaged VP40 into exosomes, and that these exosomes were capable of inducing apoptosis in recipient immune cells. Additionally, we show that presence of VP40 within parental cells or in exosomes delivered to naïve cells could result in the regulation of RNAi machinery including Dicer, Drosha, and Ago 1, which may play a role in the induction of cell death in recipient immune cells. Exosome biogenesis was regulated by VP40 in transfected cells by increasing levels of ESCRT-II proteins EAP20 and EAP45, and exosomal marker proteins CD63 and Alix. VP40 was phosphorylated by Cdk2/Cyclin complexes at Serine 233 which could be reversed with r-Roscovitine treatment. The level of VP40-containing exosomes could also be regulated by treated cells with FDA-approved Oxytetracycline. Additionally, we utilized novel nanoparticles to safely capture VP40 and other viral proteins from Ebola VLPs spiked into human samples using SDS/reducing agents, thus minimizing the need for BSL-4 conditions for most downstream assays. Collectively, our data indicates that VP40 packaged into exosomes may be responsible for the deregulation and eventual destruction of the T-cell and myeloid arms of the immune system (bystander lymphocyte apoptosis), allowing the virus to replicate to high titers in the immunocompromised host. Moreover, our results suggest that the use of drugs such as Oxytetracycline to modulate the levels of exosomes exiting EBOV-infected cells may be able to prevent the devastation of the adaptive immune system and allow for an improved rate of survival.

## Introduction

Ebola virus (EBOV), a member of the family *Filoviridae*, is an enveloped, non-segmented, single-stranded, negative sense RNA virus first recognized in 1976 (Ebola Virus Infection, 1977; Foster, 1999). EBOV can cause severe hemorrhagic fever in humans and non-human primates, resulting in high rates of morbidity and mortality (Elshabrawy et al., 2015; Singh et al., 2015). The largest and most recent outbreak of EBOV occurred in West Africa starting in August of 2014, resulting in over 28,600 cases and more than 11,300 deaths as of April 2016 according to the CDC (2016). The cellular tropism of EBOV is fairly broad, targeting mainly monocytes, macrophages, Kupffer cells and dendritic cells early in infection, helping to maintain the replication and spread of the virus (Martines et al., 2015; Messaoudi et al., 2015; Singh et al., 2015). While EBOV infection results in a massive systemic cytokine storm correlating with the poor prognosis of patients, the virus also utilizes several immune evasion strategies once inside host cells, including downregulation of cytokines and IFNα/β, dsRNA masking, inhibition of T cell activation by dendritic cells, and blocking of innate immune system signaling cascades (Messaoudi and Basler, 2015; Messaoudi et al., 2015; Rougeron et al., 2015; Singh et al., 2015).

While EBOV disease has not yet been recognized to exhibit latency, there have been several recent cases of a recurrence of infection in survivors of acute EBOV infection. Despite clearance of virus from the blood, EBOV is able to persist in immunologically protected sites in the body, including ocular fluid, semen, and vaginal fluids, as well as in sweat, urine, and breast milk (Deen et al., 2015; Varkey et al., 2015; Chancellor et al., 2016; Harries et al., 2016; MacIntyre and Chughtai, 2016). A recent study of survivors in Sierra Leone showed that EBOV may persist for much longer than previously recognized, with over 1 in 4 male EBOV survivors containing virus in their semen for up to 7–9 months post disease onset (Deen et al., 2015). Furthermore, this persistence may lead to transmission of the virus. One case involving a 44-year-old woman in Liberia in 2015 involved a potential transmission link from a 46-year-old man that had recovered from EBOV in late September of 2014. Testing of the semen by RT-PCR 199 days following the male patient’s recovery showed positive results for EBOV of the same genetic strain as the female patient (Christie et al., 2015; MacIntyre and Chughtai, 2016). The mechanism underlying viral persistence and possible recurrence in survivors is not well understood, but should be more fully investigated.

Ebola virus is composed of seven genes that encode eight proteins including: the nucleoprotein (NP); viral proteins VP24, VP30, VP35, and VP40; viral RNA-dependent RNA polymerase (L) and two possible forms of glycoprotein (GP). The EBOV GP protein is the sole protein expressed on the surface of the virus, and is essential in the attachment of EBOV to host cells (Lee and Saphire, 2009; Nyakatura et al., 2015). The mechanism of entry of EBOV into host cells is mainly via GP-dependent macropinocytosis followed by trafficking through endosomal vesicles (Nanbo et al., 2010; Saeed et al., 2010; Messaoudi et al., 2015), although the factors that direct the movement of endosomal virus vesicles are not fully understood (Sakurai et al., 2015). NP is an essential component of the nucleocapsid, and coats the viral genome prior to packaging into new infectious virions and release from the cell (Falasca et al., 2015; Messaoudi et al., 2015; Rougeron et al., 2015).

VP40 is the EBOV matrix protein and plays an essential role in driving the assembly and budding of new viral particles (Panchal et al., 2003; Silva et al., 2012; Soni and Stahelin, 2014). It forms a coating with the inner leaflet of the lipid bilayer, where it then oligomerizes in lipid rafts and recruits other host cell factors such as TSG101, Alix, and other machinery associated with the Endosomal Sorting Complex Required for Transport (ESCRT) complexes to aid in the budding and release of new virions (Martin-Serrano et al., 2001; Licata et al., 2003; Panchal et al., 2003; Silvestri et al., 2007; Han et al., 2015). VP40 alone is capable of spontaneously releasing virus-like particles (VLPs) from infected cells, which are similar in size and structure to infectious virions (Timmins et al., 2001; Bavari et al., 2002; Panchal et al., 2003). This budding phenomena is enhanced by the addition of GP and/or NP in association with VP40 (Bavari et al., 2002; Noda et al., 2002; Licata et al., 2004). Due to their morphological similarity to Ebola virions, VLPs have been utilized as a surrogate research model to study filoviruses outside of BSL-4 conditions (Kallstrom et al., 2005). Additionally, EBOV VP40 may potentially act as a suppressor of the RNA interference (RNAi) pathway, a widely conserved gene regulatory mechanism responsible for providing an innate response to viral infection through the production of microRNAs (miRNA), thereby inhibiting innate antiviral responses of the host (Fire et al., 1998; Novina and Sharp, 2004; Fabozzi et al., 2011).

In recent years, much interest has been focused on mechanisms of intracellular delivery of nucleic acids, miRNAs, and proteins between cells, particularly in the context of infections. Nanovesicles called exosomes are small membrane-bound vesicles that originate from late endosomes, which act as intercellular messengers and transport biological macromolecules from one cell to another (Théry et al., 2002; Février and Raposo, 2004; Fleming et al., 2014). Cellular proteins, as well as mRNA and miRNA, can be carried by exosomes to induce a change in recipient cells (Lotvall and Valadi, 2007; Skog et al., 2008). Additionally, exosomes from virally infected cells such as Epstein-Barr, Kaposi sarcoma-associated herpesvirus, HIV-1, and Rift Valley Fever Virus have been shown to transfer viral non-coding to neighboring uninfected cells, resulting in altered cellular activity (Pegtel et al., 2010; Barth et al., 2011; Pegtel et al., 2011; Ahsan et al., 2016; Sampey et al., 2016). If there is indeed latency in Ebola infection, it is possible that the virus could potentially utilize exosomes to transport viral nucleic acid and proteins to other neighboring cells, explaining in part the observed pathogenesis.

In this study, we examined the effect of Ebola structural proteins VP40, GP, NP and VLPs on recipient immune cells, as well as the effect of transfected cell exosomes on naïve immune cells. We found that VP40 is indeed packaged into exosomes and that these exosomes are capable of inducing programmed cell death in recipient immune cells. Additionally, we show that presence of VP40 within cells or contained in exosomes can result in an altered expression of RNAi components in host cells. Presence of VP40 in cells induced an upregulation of extracellular CD63 and Alix, as well as ESCRT-II proteins EAP20 and EAP45, indicating that VP40 may be capable of increasing exosomal biogenesis. We also found that VP40 is phosphorylated by Cdk2/Cyclin E and Cdk2/Cyclin A complexes at the G1/S border and can be reversed with Cdk2 inhibitors. Finally, we show that specific nanoparticles are able to capture and detect Ebola proteins present in human samples under BSL-2 conditions, potentially representing a novel diagnostic tool that could easily be used in the field. Overall, our data indicates that the VP40 packaged into exosomes may be responsible for the bystander lymphocyte apoptosis and dysregulation of the immune system observed during pathogenesis, allowing for high levels of viral replication in immunocompromised infected individuals.

## Materials and Methods

### Cell Culture and Reagents

CEM, Jurkat and U937 cells were obtained from ATCC (Manassas, VA, United States) and maintained in RPMI-1640 media containing 10% heat-inactivated fetal bovine serum (FBS), 1% L-glutamine, and 1% streptomycin/penicillin (Quality Biological, Gaithersburg, MD, United States). Monocytic U937 cells were induced to differentiate into monocyte-derived macrophages (MDM) with 50 nM of phorbol-12-myristate-13-acetate (PMA; Sigma–Aldrich) for 5 days. The primary monocytes were isolated from peripheral blood mononuclear cells (PBMCs; purchased from Precision Biosciences) and maintained in RPMI-1640 media as described above. Primary monocytes isolated from PBMCs were induced to differentiate into MDMs with 10 nM of phorbol-12-myristate-13-acetate (PMA) for 2 days. HEK-293T cells were maintained in Dulbecco’s modified minimum essential medium (DMEM) media containing 10% heat-inactivated FBS, 1% L-glutamine, and 1% streptomycin/penicillin (Quality Biological, Gaithersburg, MD, United States). All cells were incubated at 37°C in the presence of 5% CO_2_.

For antibiotic selection of transfected 293T cells, Hygromycin B (Invitrogen), Zeocin (Life Technologies), or Geneticin (Life Technologies) were added to the culture next day. Hydroxyurea (Sigma–Aldrich) and r-Roscovitine (Cell Signaling Technology) were used for the treatment of transfected cell cultures for *in vivo* labeling followed by kinase assay. Other Cdk2 inhibitors used for kinase assays (Alsterpaullone, Indirubin-3′-monoxime, and Purvalanol A) were purchased from Sigma–Aldrich. Treatment of transfected 293T cells with Oxytetracycline (Selleck Chemicals), Esomeprazole (Selleck Chemicals), and Cambinol (Sigma–Aldrich) for analysis of levels of exosomal markers took place the day following transfection. All experiments involving biohazards were carried out under the IBC-approved institutional biosafety guidelines and were performed at BSL-2 level.

### Plasmids, Transfections, and Generation of Resistant Clones

Ebola structural proteins were expressed from plasmids (Invitrogen) with CMV promoters and specific antibiotic selection markers: GP (pcDNA3.1/Zeo), NP [pcDNA3.1 (±)], VP40 (pcDNA3.1/Hygro). Twenty microgram of *Escherichia coli*-purified DNA was transfected into 293T cells using either Attractene reagent (Qiagen, Chatsworth, CA, United States) according to the manufacturer’s instructions, or by electroporation as previously described (Kashanchi et al., 1992). Transfected cells were treated next day with either 100 μg/mL Zeocin (CMV-GP), 500 μg/mL Geneticin (CMV-NP), or 200 μg/mL Hygromycin B (CMV-VP40) for plasmid selection. To generate resistant clones, transfected cells were cultured for >2 weeks, followed by isolation of surviving colonies (<1% of cells) and multiple passages under specific antibiotic selection. A total of 13 resistant clones were generated, but only 2 survived after >10 passages: one GP-clone (data not shown) and one VP40-clone (EVTR2C). All resistant clones were kept under constant antibiotic selection.

### Capture of Exosomes, Ebola VLPs, and Proteins with Nanotrap Particles

The nanotraps utilized here are multifunctional hydrogel particles 700–800 nm in diameter composed of high affinity aromatic baits containing a core surrounded by a sieving shell with pores that will selectively allow the passage of smaller molecules. Polymerization reactions with reactants *N*-isopropyl acrylamide (NIPAm), *N,N*-Methylenebisacrylamide (Bis) and either allylamine (AA), acrylic acid (AAc) or methacrylate (MA) are required for the synthesis of the particle’s shell. Vinyl sulfonic acid (VSA) monomers may also be incorporated into the shell for greater exclusion of high molecular weight and highly abundant analytes. Specific high affinity charge-based baits are covalently attached to the core matrix and bind target molecules through hydrophobic and electrostatic interactions, preventing degradation while sequestering the targets. NT80 beads contain Reactive Red 120 core bait while NT82 beads have Cibacron Blue F3GA. Both bead types have a NIPAm-Bis-AA matrix with a shell lacking VSA (Jaworski et al., 2014b). The characteristics of the newer nanotrap particles utilized in this manuscript are outlined in **Figure 3B**. For the capture and isolation of exosomes from transfected cell culture supernatants, a 20 μL slurry (30%) of 1:1 NT80 and NT82 nanotrap particles (Ceres Nanosciences) was incubated with 500 μL of cell-free supernatant. For capture of exosomes from resistant clone cell culture supernatants, a 25 μL slurry (30%) of NT80/82 beads (1:1) was incubated with 1 mL of cell-free, filtered (0.22 micron) supernatant. Samples were bound at 4°C for 72 h. NT pellets were isolated and washed with 500 μL of sterile 1x PBS without calcium and magnesium, followed by resuspension in 20 μL of Laemmli buffer for SDS/PAGE. For the capture and isolation of VP40, 69 ng of *E. coli*-purified VP40 was spiked into 100 μL of PBS and incubated with 20 μL of slurry (30%) of nine different NTs (210, 212, 213, 217, 219, 222, 223, 224, and 229) overnight at 4°C. Beads were washed with sterile 1x PBS without calcium and magnesium and resuspended in 20 μL Laemmli buffer for SDS/PAGE. For the capture and isolation of VLP in the presence of SDS, 20 μL of 30% slurry of the 9 NTs listed above was incubated with 1 μg of purified VLP in 80 μL SDS buffer [1:4; 20 μL Laemmli buffer and 60 μL Radioimmunoprecipitation assay (RIPA) buffer] overnight at 4°C. NT pellets were washed once with 500 μL of TNE50 + 0.1% NP40, followed by resuspension in Laemmli buffer for SDS/PAGE. For capture and isolation of VLP-derived VP40 in human samples, 100 μL of healthy human saliva (BioreclamationIVT, Baltimore, MD, United States), urine (obtained from a healthy male lab worker), and serum (#AY217; NCI bank transferred to FK) from healthy anonymous donors were diluted 1:3 in sterile 1x PBS without calcium and magnesium and spiked with 2.5 μg of purified Ebola VLP. One hundred and fifty microliters each of RIPA and Laemmli buffer (total 300 μL of 1:1 SDS buffer) was added to each sample, followed by addition of 60 μL of a 30% slurry of NT219. Samples were incubated overnight at 4°C, washed once with 500 μL of TNE50 + 0.1% NP40, and resuspended in 20 μL of Laemmli buffer for SDS/PAGE.

### Cell Treatment and Viability Assay

Cells were seeded into 96 well plates at 50,000 cells per well in fresh media followed by treatment. For treatment of CEM, Jurkat, and U937 cells with VLP or VP40, 0.1 or 0.5 μg of VLP or *E. coli*-purified VP40 (generously provided by IBT Bioservices) was placed into 100 μL of cells suspended in fresh media. For treatments with cell culture supernatants, transfected cells or resistant clones were cultured for 5 days to maximize the concentration of exosomes within the media as previously described (Narayanan et al., 2013). Media was then harvested from the cells, centrifuged for 10 min at 14,000 rpm to remove cells and cellular debris, and either filtered (0.22 micron) or left unfiltered. Seeded cells in fresh media (50 μL total) were then treated with 50 μL of supernatant. All treatments of CEM, Jurkat, and U937 cells were allowed to incubate for 5 days followed by measurement of cell viability using CellTiter-Glo Cell Luminescence Viability kit (Promega, Madison, WI, United States) as per manufacturer’s instructions. Briefly, 100 μL CellTiter-Glo reagent was added to the wells (1:1 reagent:cell suspension) and incubated at room temperature for 10 min followed by detection of luminescence using the GLOMAX multidetection system (Promega). Primary macrophages were assayed for cell viability 2 days following treatment.

### Preparation of Whole Cell Extracts, Western Blot Analysis, and Densitometry

Cell pellets were harvested, washed twice with 1x PBS without calcium and magnesium, and resuspended in lysis buffer [50 mM Tris-HCl (pH 7.5), 120 mM NaCl, 5 mM EDTA, 0.5% Nonidet P-40, 50 mM NaF, 0.2 mM Na_3_VO_4_, 1 mM DTT, and 1 complete protease inhibitor mixture table/50 mL (Roche Applied Science, Mannheim, Germany)]. The suspension was incubated on ice for 20 min with gentle vortexing every 5 min, followed by centrifugation at 10,000 rpm at 4°C for 10 min. Protein concentration from the lysate supernatant was determined using the Bradford protein assay (Bio-Rad).

Whole cell extracts (10 μg) were resuspended in 10 μL Laemmli buffer, heated at 95°C for 3 min, and loaded onto a 4–20% Tris-glycine SDS gel. Nanotrap particle pellets were resuspended in 15 μL Laemmli buffer, heated at 95°C for 3 min and vortexed three times until fully resuspended, and then loaded onto a 4–20% Tris-glycine SDS gel. Gels were run at a maximum 160 V and transferred overnight onto PVDF membranes. Membranes were blocked in 5% milk in 1x PBS containing 0.1% Tween 20 for 1 h at 4°C, then incubated overnight at 4°C with appropriate primary antibody: [α-Caspase 3, α-PARP-1, α-Alix, α-CHMP6, α-TSG101, α-EAP20, α-EAP45, α-TRBP (Santa Cruz Biotechnology); α-Dicer, α-Drosha, α-Ago1, α-Exportin 5, α-DGCR8, α-CD63, α-β-actin, (Abcam); α-VP40 and α-GP] were generously provided by IBT Bioservices. Membranes were then incubated with the appropriate HRP-conjugated secondary antibody for 2 h at 4°C and developed using SuperSignal West Dura Extended Duration Substrate (Pierce, Rockford, IL, United States). Luminescence was visualized on a Kodak 1D image station (Carestream Health, Rochester, NY, United States). Raw densitometry counts were obtained using ImageJ software.

### Identification of Potential Phosphorylation Sites in VP40 Protein

Mass spectra for VP40 was obtained for this manuscript as previously described in our analysis of VP30 from EBOV virions (Ilinykh et al., 2014). Proteomics data for VP40 had not been previously published. The spectra were analyzed by SEQUEST (Thermo) search engine. Protein phosphorylation sites were detected with Proteome Discoverer 1.4 software and mapped to the VP40 protein sequence.

### Immunoprecipication, *in vivo* Labeling, and Kinase Assays

Immunoprecipitation (IP) was performed by incubation of 500 μg of CEM or transfected and treated 293T whole cell extracts with 10 μg of appropriate primary antibody (α-Cdk2, α-CycE, α-CycA, α-normal rabbit IgG; Santa Cruz Biotechnology) and 100 μL TNE50 + 0.1% NP-40 for 48 h at 4°C. CEM cells were utilized for these experiments as we have previously shown that these cells contain active Cdk/Cyclin complexes that can easily be purified using specific antibodies (Wang et al., 2001). The next day, complexes were precipitated with 30 μL of a 30% slurry of A/G beads (Calbiochem) for 2 h at 4°C, washed twice with TNE50 + 0.1% NP-40 and twice with kinase buffer. The reaction mixtures (20–30 μL) contained the following final concentrations: 40 mM β-glycerophosphate (pH 7.4), 7.5 mM MgCl_2_, 7.5mM EGTA, 5% glycerol, [γ-^32^P] ATP (0.4 mM, 1 μCi), 50 mM NaF, 1 mM orthovanadate, and 0.1% (v/v) β-mercaptoethanol. Phosphorylation reactions were performed with immunoprecipitated material and *E. coli*-purified VP40 (0.5 μg) as a substrate in TTK kinase buffer containing 50 mM HEPES (pH 7.9), 10 mM MgCl_2_, 6 mM EGTA, and 2.5 mM dithiothreitol. Reactions were incubated at 37°C for 1 h, stopped by the addition of 1 volume of Laemmli sample buffer containing 5% β-mercaptoethanol, and run on 4–20% SDS-polyacrylamide gel. Gels were subjected to autoradiography and quantification using PhosphorImager software (Amersham Biosciences). Kinase assay with peptides was accomplished by adding 50 μg appropriate peptide [S3 (ADLTSPEKIQAI), S3M (ADLTAPEKIQAI), T7 (GKKVTAKNGQPI), S8 (GKKVASKNGQPI), T2 (LVHKLTGKKV) (Biomatik USA, LLC, Wilmington, DE, United States)] and 15 μL of appropriate antibody (α-CycE, α-IgG) to the Protein A/G pellet suspended in kinase buffer (120 μL). Next, [γ-^32^P] ATP was added to the samples, incubated at 37°C for 1 h, and samples (10 μL) were dotted onto Whatman glass microfiber filters, dried for 30 min, and subsequently submerged in TE buffer with gentle agitation for 48 h. Samples were quantified using a scintillation counter (QuantaSmart^TM^ DPM assay count).

For *in vivo* labeling, 293T cells (5 × 10^6^) were electroporated with 20 μg of VP40 plasmid, followed by addition of Hygromycin B (200 μg/mL). Cells were grown up to 30–40% confluency (4 days) at which time Hydroxyurea (G1/S blocker; 1 mM) was added for one additional day. Media were removed and 1 mL of DMEM was added to cover the cells, with the addition of 10 μL of [γ-^32^P] ATP (∼3000 mCi/mL) for 4 h. Next, r-Roscovitine (1–10 μM) was also added to a few of the samples. After labeling, cells were chased with cold complete media (no radioactivity) for 2 h. Cells were removed with a cell scraper and lysed in lysis buffer, followed by IP with α-VP40 antibody overnight in TNE150 + 0.1% NP-40. Protein A/G was added and bound beads were washed 2x with TNE150 + 0.1% NP-40 and once with kinase buffer. Pellets were then resuspended in Laemmli buffer and run on 4–20% SDS-polyacrylamide gel. Gels were subjected to autoradiography and quantification using PhosphorImager software (Amersham Biosciences).

### Isolation of Exosomes and AChE Assay

293T, transfected 293T, and EVTR2C cells were grown in DMEM containing 10% heat-inactivated FBS, 1% L-glutamine, and 1% streptomycin/penicillin (Quality Biological, Gaithersburg, MD, United States). Exosome preparation was made from 100 mL of cell culture supernatant (produced from fully confluent cells grown for 5 days). Cells were pelleted by centrifugation at 300 *g* for 10 min. An additional centrifugation at 2000 *g* for 10 min was used to pellet dead cells. The resulting supernatant was then filtered through a 0.22 micron filter and ultracentrifuged at 10,000 *g* for 30 min. This resulting supernatant was then pulled off and ultracentrifuged at 100,000 *g* for 70 min to pellet the exosomes. These exosomes were then washed with 22.5 mL of sterile 1x PBS without calcium and magnesium and ultracentrifuged again at 100,000 *g* for 70 min. The supernatant was pulled off and the final pellet was resuspended in 100 μL of PBS. All spins were performed at 4°C. Protein levels of the exosomes were determined with the Amplex^®^ Acetylcholine/Acetylcholine Esterase Activity Assay Kit (Thermo A12217) following the manufacturer’s instructions. Briefly, a 1x running buffer negative control (20 mL of H_2_O and 5 mL of 5x reaction buffer) and two positive controls, one consisting of acetylcholine esterase and one consisting of hydrogen peroxide, were made and plated on a 96-well plate. Exosomes were treated and fluorescence of acetylcholine esterase activity was measured with a GLOMAX multidetection system (Promega) every 15 min for 1 h to find optimal activity.

### Statistical Analysis

Standard deviation was calculated in all quantitative experiments done in triplicate. All *p*-values were calculated using two-tailed Student’s *t*-tests (Microsoft Excel) and were considered to be statistically significant when *p* < 0.05.

## Results

### Treatment of Immune Cells with Ebola VLP or VP40 Deregulates Cell Growth

**FIGURE 1:**
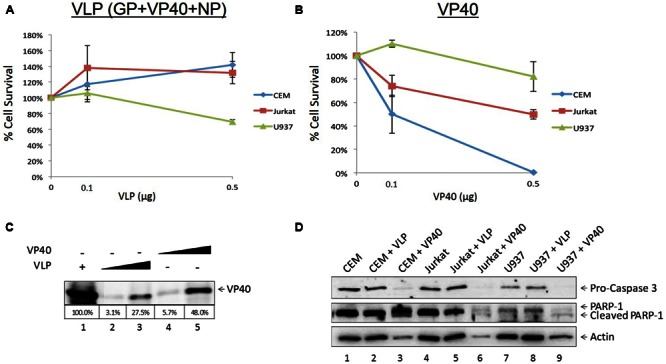
**Treatment of recipient cells with EBOV VLPs and free VP40.** Mid log phase CEM, Jurkat (T-cells) and U937 (monocyte) cells (5 × 10^4^) were treated with either Ebola VLPs containing GP, NP, and VP40 **(A)** or *E. coli*-purified VP40 protein **(B)** in concentrations of 0.1 or 0.5 μg and incubated for 5 days, followed by cell viability assay with CellTiter-Glo. **(C)** VLP and purified VP40 proteins (0.1 or 0.5 μg) were run on a 4–20% SDS/PAGE and analyzed by Western blot for levels of VP40. Percentage of VP40 with 5 μg purified VLP positive control set to 100% was determined by densitometry and is shown in the bottom panel. **(D)** Two hundred microliters of CEM, Jurkat, and U937 cells (5 × 10^5^) was treated with 1 μg of Ebola VLP or *E. coli*-purified VP40 protein and incubated for 5 days. Cells were lysed, run on a 4–20% SDS/PAGE, and analyzed by Western blot for the presence of apoptotic markers Pro-Caspase 3, cleaved and un-cleaved PARP-1, and Actin.

It has been well documented that EBOV as well as Ebola VLPs are capable of inducing significant cell death as well as cytokine storm *in vitro* and *in vivo* (Wauquier et al., 2010; Wahl-Jensen et al., 2011; Falasca et al., 2015; Messaoudi and Basler, 2015), particularly in macrophages and dendritic cells. Additionally, bystander lymphocyte apoptosis has been observed in many experimental and clinical models of Ebola infection (Geisbert et al., 2003; Wauquier et al., 2010). However, direct cell regulation of lymphocytes has not been studied by Ebola viral VLPs or structural proteins. We hypothesized that one or more of EBOV proteins may cause deregulation of cell growth on recipient immune cells. To this end, we incubated increasing concentrations of VLP and VP40 with three immune cell types, including two types of T-cells (early and late developmentally deregulated cells represented by CEM and Jurkat cells, respectively) and one monocyte cell line (U937), and assayed for cell viability using CellTiter-Glo. Purified *E. coli*-made VP40 was utilized, as the other two proteins GP and NP also found in VLPs were insoluble using prokaryotic vectors (data not shown). Only two concentrations of VLP and VP40 were used for these experiments due to limited availability of reagents. Results in **Figure 1A** show that VLPs (at varying concentrations) were not apoptotic in lymphocytes, while monocytes showed a slight decrease in cell viability at higher VLP concentrations. On the other hand, when using *E. coli*-purified VP40, the two T-cell lines showed a decrease in viability (**Figure 1B**). To verify the relative levels of VP40 in the purified VP40 versus that found in the VLPs, assessment by Western blot showed a slightly higher amount of VP40 (approximately 1.75-fold higher levels of VP40 compared to VLP, as determined by densitometry analysis) in the *E. coli*-purified preparation than that in VLPs (**Figure 1C**). Importantly, VP40 within VLPs is likely to be hidden under the envelope, therefore only the unmasked VP40 decreased T-cell viability shown in **Figure 1B**. We have recently shown that analysis of apoptotic markers Caspase 3 and PARP-1 by Western blot in the case of Rift Valley Fever virus infection indicated cell death by apoptosis in Jurkat cells treated with exosomes containing proteins from RVFV resistant clones (Ahsan et al., 2016). Similarly, here T-cells and monocytes treated with VP40 showed increased pro-Caspase cleavage and accumulation of cleaved PARP-1 (**Figure 1D**), confirming that cells treated with free VP40 protein initiated programmed cell death. The levels of Actin in the VP40-treated lanes (lanes 3, 6, and 9) decrease as well, which is also indicative of cell death. Collectively, these results indicate that free VP40 can induce apoptosis in immune cells, especially in T-cells.

### Treatment with Ebola Structural Protein-Transfected Cell Supernatants Deregulates Immune Cell Growth

**FIGURE 2:**
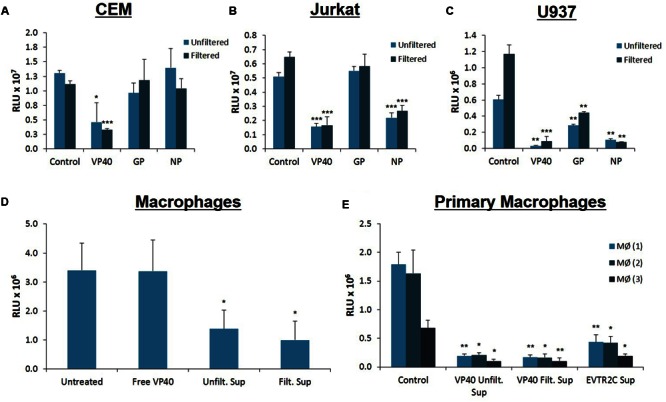
**Treatment of immune cells with filtered and unfiltered Ebola-transfected cell supernatants.** 293T cells were transfected with either VP40, GP, or NP plasmids with Attractene Transfection Reagent (Qiagen) as per the manufacturer’s instructions and incubated for 2 days. Transfection complexes were removed and cells were treated with antibiotics for plasmid selection for 14 days. Transfection supernatants were either left unfiltered or passed through a 0.22 micron filter. Control 293T cells were not transfected nor treated with antibiotics (Hygromycin B, Zeocin, or Geneticin). Seventy-five microliters (0.45 mU AChE) of both filtered and unfiltered supernatants were used to treat CEM **(A)**, Jurkat **(B)** and U937 **(C)** cells, which were subsequently assayed for cell viability via CellTiter-Glo after 4 days of incubation. Statistical significance was determined using 2-tailed student’s *t*-test, with filtered or unfiltered experimental values compared to the Control values of like type. **(D)** U937 cells were treated with PMA (50 nM) for 5 days to stimulate differentiation into macrophages. On day 5 media was replaced and differentiated U937 macrophages were treated with either *E. coli*-purified free VP40 protein, or supernatants from VP40-transfected 293T cells (filtered and unfiltered). Untreated macrophages received no external supernatant treatment. Cells were incubated for an additional 5 days and cell viability was assayed with CellTiter-Glo. **(E)** Primary monocytes from 3 healthy donors were treated with PMA (10 nM) for 2 days to stimulate differentiation into macrophages. Primary macrophages were then treated with supernatants from either VP40-transfected 293T cells (filtered and unfiltered), or from VP40-resistant clone EVTR2C cells (filtered). Control primary macrophages were treated with filtered supernatant from normal 293T cells. Cells were incubated for 2 days before being assayed for viability with CellTiter-Glo. Statistical significance was determined using 2-tailed student’s *t*-test, with filtered or unfiltered experimental groups compared to the Control groups of like type. MØ, Macrophage; RLU, Relative Luminescent Units. ^∗^*p* < 0.05, ^∗∗^*p* < 0.01, ^∗∗∗^*p* < 0.001.

Ebola-infected cells are capable of production and release or accumulation of EBOV proteins in a cell type dependent manner (Steele et al., 2001). As we have previously shown dysregulation of immune cells by supernatants from HIV, HTLV, and Rift Valley Fever virus infected cells (Narayanan et al., 2013; Jaworski et al., 2014a; Ahsan et al., 2016; Sampey et al., 2016), we wished to determine whether supernatants from cells transfected with Ebola structural protein-containing plasmids would impact the viability of neighboring uninfected cells. We transfected 293T cells with VP40, GP, and NP plasmids followed by specific antibiotic selection for each plasmid. The supernatants from the transfected cells were isolated and used to treat uninfected CEM, Jurkat, and U937 cells. Cell viability was assayed 5 days following treatment using CellTiter-Glo. Statistical analyses were performed comparing filtered supernatants from transfected cell to filtered supernatants from control 293T cell treatments, and likewise for the unfiltered groups. As shown in **Figure 2A**, only CEM cells treated with supernatants from VP40-transfected 293T cells showed significant reduction in viability. However, supernatants from NP- or GP-transfected cells had minimal effect on CEM cell viability. Jurkat T-cells showed significant decreases in viability following treatments with VP40 and NP transfection supernatants in contrast to treatment with GP-transfected cell supernatants (**Figure 2B**). Interestingly, CEM cells are derived from the T lymphoblast whereas Jurkat cells originate from the more differentiated peripheral blood T lymphocyte. As such, the differential dysregulation seen between these two cell types may be an indication of selective susceptibility of T cells to Ebola structural proteins based on their stage of development. Finally, U937 monocytic cells showed significant reduction in viability when treated with any of the three supernatants, with VP40-transfected supernatants showing the most pronounced effect (**Figure 2C**). These results suggest that monocytes, in contrast to T cells, are particularly susceptible to Ebola protein-transfected cell supernatants. When CEM, Jurkat, and U937 cells were treated with supernatants from 293T cells that were transfected with multiple plasmid combinations (i.e., VP40 + GP, VP40 + NP, GP + NP, and VP40 + GP + NP), all three immune cell types showed significant decreases in viability from the triple combination of VP40 + GP + NP while only the Jurkat cells showed significant decreases from treatment with supernatants from all transfection doublets (data not shown). Furthermore, antibiotic alone (Hygromycin B, Geneticin, and Zeocin) treated supernatants of 293T cells (5 days) did not alter cell viability of immune cells (data not shown).

As VP40 alone or VP40 + GP ± NP can form VLPs, we next asked whether the specific size of the material in supernatants of transfected cells contributed to the observed decrease in viability. VLPs are distinct from other extracellular vesicles, which can be differentiated based on a number of characteristics, including size and surface markers. VLPs are 400 nm in size or larger, similar to EBOV virions (Bavari et al., 2002; Noda et al., 2002; Warfield et al., 2003), whereas extracellular vesicles (i.e., exosomes) are smaller than 220 nm, and typically approximately 100 nm (Fleming et al., 2014; Sampey et al., 2014). For this reason, we passed transfected cell supernatants through a 0.22 micron filter and asked whether the same phenotype in treated immune cells could be observed compared to those treated with unfiltered supernatants. A side-by-side comparison of filtered versus unfiltered supernatants showed similar effects on all cell types (**Figures 2A–C**). Since filtering did not abrogate the earlier observed effects, we concluded that it was unlikely that the detrimental agent within the transfected cell supernatant was Ebola VLPs or cellular apoptotic bodies that are also larger than 400 nm (Akers et al., 2013; Schwab et al., 2015). Additionally, these experiments indicated that the factor(s) effecting the apoptosis was not free VP40 protein, as purified VP40 showed little effect on U937 cell viability (**Figure 1B**). Collectively, these results imply that supernatants from Ebola protein-transfected 293T cells may be capable of negatively impacting both T cells and monocytes.

As the supernatants from cells transfected with VP40 DNA appeared to pose greater toxicity to monocytes than to T lymphocytes, we wished to explore further the effect of VP40 on differentiated macrophages. This was of interest since EBOV naturally targets monocytes and especially macrophages *in vitro* and *in vivo* during early infection (Jaax et al., 1996; Ryabchikova et al., 1999; Geisbert et al., 2003; Falasca et al., 2015; Messaoudi and Basler, 2015; Messaoudi et al., 2015; Nyakatura et al., 2015; Singh et al., 2015). We differentiated U937 monocytes into macrophages using phorbol 12-myristate 13-acetate (PMA) for 5 days, followed by treatment with purified VP40 protein, as well as unfiltered and filtered VP40 transfection supernatants. Differentiated cells were incubated for 5 days and cell viability was measured using CellTiter-Glo. While purified VP40 protein alone had no effect on differentiated U937 cell viability, both filtered and unfiltered cell-free media from VP40-transfected cells resulted in significantly decreased cellular viability (**Figure 2D**).

Next, we differentiated primary monocytes into macrophages using PMA for 2 days and treated them with filtered supernatant from control 293T cells, unfiltered or filtered supernatants from VP40-transfected 293T cells, or filtered supernatants from Ebola VP40-Transfected Resistant 293T Clone (EVTR2C) cell supernatants. EVTR2C cells are a clone of VP40-transfected 293T cells resistant to the effects of VP40. These resistant clones are a stable population of 100% VP40-transfected 293T cells under Hygromycin B selection. We believe that EVTR2C cells are able to survive stably with the production of a lower level of VP40 protein in comparison to normal VP40-transfected 293T cells (see Materials and Methods). Differentiated primary cells were incubated for 2 days and cell viability was measured using CellTiter-Glo. The primary cells were only treated for 2 days with cell supernatants as opposed to the 5-day treatment received by the U937 cells. Viability of the treated primary cells was noticeably decreasing over time upon visual inspection. Strikingly, PMA-activated primary monocyte viability was drastically reduced with the treatment of unfiltered and filtered VP40-transfected 293T and filtered EVTR2C cell supernatants in comparison to the control treatment (**Figure 2E**). This further confirms that free, purified VP40 protein alone exhibits no toxicity to monocytes; however, primary monocytes that have differentiated into macrophages are more susceptible to supernatant from VP40-producing cells, compared to macrophages derived from monocyte cell lines. Collectively, these data suggest that VP40 produced from transfected cells can potentially be packaged into protected vesicles, which in turn can exert detrimental effects on immune recipient cells.

### Exosomes from Transfected Cells Contain Ebola VP40

**FIGURE 3:**
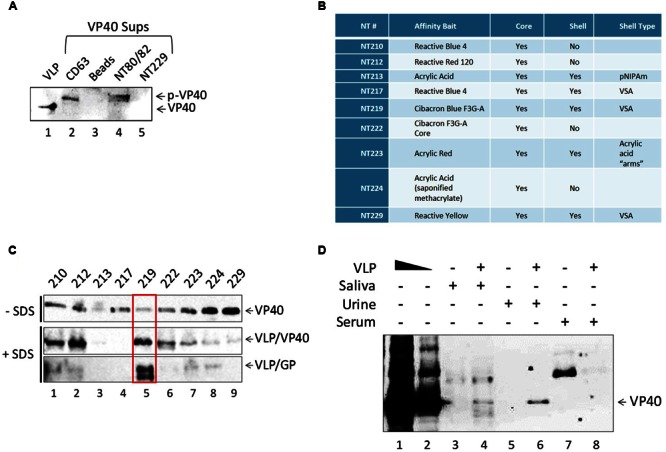
**Detection of free and exosome-associated VP40 using nanoparticles. (A)** Five hundred microliters of VP40-transfected supernatant (1.75 units of AChE) was incubated with either CD63 beads, beads alone, NT80/82 or NT229 beads. Samples were bound at 4°C for 72 h. Pellets were isolated and washed once with 1x PBS, followed by Western blot for VP40. Control input contained 0.9 μg of Ebola VLP (GP + VP40 + NP) in Laemmli buffer. **(B)** The affinity bait, core, shell, and corresponding shell type of all nanotrap particles used. **(C)** One hundred nanograms of *E. coli*-purified VP40 were added to 20 μL of each nanoparticle 30% slurry in 100 μL PBS. Binding occurred overnight at 4°C, beads were washed and resuspended in Laemmli buffer, and samples were run on a 4–20% Tris-glycine SDS gel. Western blot was performed with a 1:1000 dilution of α-VP40 antibody incubated overnight, three PBS washes and incubation with 3 μL of α-rabbit secondary antibody for 2 h, followed by development. Alternatively, 1 μg of purified Ebola VLP was added to 20 μL of each nanoparticle 30% slurry in SDS buffer (a combination of 20 μL Laemmli buffer and 60 μL of RIPA buffer). Samples were incubated overnight at 4°C, beads were washed once with TNE50 + 0.1% NP40, and pellet was resuspended in Laemmli buffer. Samples were run on a 4–20% Tris-glycine SDS gel followed by Western blot with a 1:1000 dilution of α-VP40 antibody or 1:5000 dilution of α-GP antibody. Membranes were incubated overnight, treated with 3 μL of α-rabbit secondary antibody for 2 h, and developed. **(D)** One hundred microliters of healthy human saliva, urine, and serum was diluted 1:3 in PBS and spiked with 2.5 μg of Ebola VLP. One hundred and fifty microliters each of RIPA and Laemmli buffer (a total of 300 μL of 1:1 SDS buffer) was added to each sample followed by the addition of 60 μL of 30% slurry of NT219 and rotation overnight at 4°C. Beads were spun down the next day, washed once with TNE50 + 0.1% NP40, and resuspended in Laemmli buffer followed by SDS/PAGE and Western blot for the presence of VP40. VLP positive controls were loaded at 0.5 and 0.1 μg.

We next asked whether VP40 produced in the transfected cells was incorporated into exosomes, potentially affecting recipient cells. The packaging of viral proteins into exosomes has been previously shown in many other infections including HIV-1, HTLV-1 and Rift Valley fever viruses (Meckes et al., 2013; Fleming et al., 2014; Jaworski et al., 2014a; Sampey et al., 2014; Ahsan et al., 2016). Incorporation into exosomes protects the packaged proteins and nucleic acids from degradation by extracellular enzymes and allows them to reach recipient cells. Therefore, we concentrated vesicles from the supernatants of transfected cells using CD63 Dynabeads and NT80/82 nanotrap particles (NTs), which have previously been shown to effectively capture exosomes from dilute supernatants (Jaworski et al., 2014a,b; Sampey et al., 2016). CD63 is a tetraspanin marker abundantly expressed on exosomes and also within virally infected cells (Sampey et al., 2016). Additionally, we used Dynabeads alone as a negative control, and NT229 beads which we had observed to be a strong binder of free VP40 protein (see data in **Figure 3C**). Filtered supernatants from VP40-transfected 293T cells were treated with nanoparticle beads, washed, resuspended in SDS Laemmli buffer, and analyzed by Western blot for the presence of VP40 protein. VP40 was present in the samples immunoprecipitated with CD63 and NT80/82 beads (**Figure 3A**, lanes 2 and 4), but was not precipitated with Dynabeads alone, or with NT229 beads that capture purified VP40 (**Figure 3A**, lanes 3 and 5). It is interesting to note that the VP40 from exosomes exhibited a slightly higher molecular weight, indicating selective incorporation of post-translationally modified VP40 (i.e., phosphorylation; p-VP40) into exosomes.

We have previously shown that specific nanoparticles can capture viral proteins, i.e., HIV-1 Tat (Narayanan et al., 2013; Jaworski et al., 2014b). Thus, we asked whether free VP40 could be captured with any of the newer set of 9 particles or nanotraps described in **Figure 3B**. We hypothesized that there could be free VP40, exosome-associated VP40, VLP-associated VP40, or virally associated VP40 that could be captured using these nanoparticles, presenting a potential diagnostic tool of EBOV disease progression. We investigated this by treating purified VP40 protein with nine different nanotraps (NTs 210, 212, 213, 217, 219, 222, 223, 224, and 229), some of which were previously shown to effectively capture HIV-1 viral proteins (Narayanan et al., 2013; Jaworski et al., 2014a,b), under two different binding conditions: a phosphatases-buffered saline (PBS) buffer that contained no SDS, and a second buffer that contained SDS (1:4; Radioimmunoprecipitation assay (RIPA) and Laemmli buffer). When using PBS alone, almost all particles, except NT213, showed varying levels of bound VP40, with the NT229 being the strongest binder of free VP40 (**Figure 3C**). However, when we used SDS buffer, we allowed the nanotraps to bind to VLPs in a mixture of RIPA and Laemmli buffer overnight at 4°C. The next day, samples were washed, isolated on a 4–20% SDS/PAGE, and subsequently analyzed by Western blot for VP40 and GP proteins. The bottom panels in **Figure 3C** show that in the presence of SDS buffer, NT219 was the strongest binder of Ebola proteins from VLP, whereas NT229 showed minimal or no binding in this assay.

We finally asked whether VLPs spiked into bodily fluid could be captured with NT219 beads under these strong denaturing conditions. To that end, we spiked human saliva, urine, and serum with VLPs and treated the samples with SDS buffer (1:1; Laemmli to RIPA). A 30% slurry of NT219 beads was added, samples were incubated overnight at 4°C, washed with TNE50 + 0.1% NP40 the next day and analyzed by Western blot for presence of VP40 protein. As shown in **Figure 3D**, NT219 beads were able to bind to VP40 in both saliva and urine samples under these reducing conditions. The VP40 from saliva samples contained various degraded smaller forms, whereas the urine sample showed a single band for the protein (**Figure 3D**, lanes 4 and 6). The binding efficiency was approximately 1% under these conditions as determined by densitometry (data not shown). Interestingly we observed no VP40 present from the serum samples spiked with the VLP, indicating that the proteins may have been rapidly degraded even when using SDS buffer. Collectively, these data indicate that exosome-associated VP40 can be captured with either CD63 beads or nanoparticles from transfected supernatants, and that the use of nanotraps in an SDS buffer (to disrupt VLPs, and by analogy virions) may be an alternative method in concentrating inactive Ebola proteins or potentially nucleic acids for downstream diagnostic assays.

### Ebola VP40 Is Phosphorylated by Cyclin-Dependent Kinase 2

**FIGURE 4:**
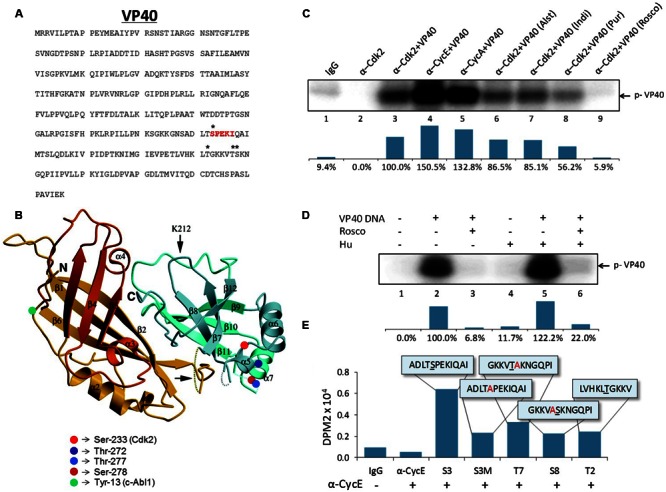
**Potential activity and sites of Cdk2/Cyclin phosphorylation on Ebola VP40. (A)** The amino acid sequence of Ebola VP40 with potential phosphorylation sites (^∗^) and hypothesized Cdk2 phosphorylation site (red). **(B)** Crystal structure of EBOV VP40 monomer. Potential phosphorylation sites are marked on two serine residues (red) and two threonine residues (blue). Previously demonstrated c-Abl1 phosphorylation site tyrosine-13 (García et al., 2012) is indicated (green). The Ebola VP40 crystal structure modified was from Dessen et al. (2000). **(C)** CEM extracts were used for immunoprecipitation using α-Cdk2, α-Cyclin E, α-Cyclin A or IgG as control. Five hundred micrograms of CEM whole cell extract was used with 10 μg of each antibody and 100 μL TNE50 + 0.1% NP-40 for 48 h at 4°C, followed by next day addition of Protein A/G for 2 h at 4°C. The complexes were washed with twice with TNE50 + 0.1% NP-40 and twice with kinase buffer, and successively used for *in vitro* kinase assay using purified VP40 protein (lanes 3–9) and [γ-^32^P] ATP. Cdk inhibitors Alsterpaullone, Indirubin-3′-monoxime, Purvalanol A and r-Roscovitine (lanes 6–9) were used at final 1 μM concentration for 1 h at 37°C. Densitometry analysis of kinase activity as a percentage is shown in the bottom panel with α-Cdk2+VP40 set to 100%. **(D)** 293T cells were transfected with 20 μg of VP40 DNA using electroporation and were placed under antibiotic selection (Hygromycin B). After 4 days, cells (30–40% confluent) were either untreated or blocked at G1/S phase of cell cycle with Hydroxyurea (1 mM) for 1 day. Cells were subsequently radio-labeled with [γ-^32^P] ATP and a few samples were treated with r-Roscovitine (10 μM) for 4 h in complete media. Radioactive material was subsequently removed, washed and chased with complete media for 2 h. Next, cells were removed (cell scraper), lysed with lysis buffer, and immunoprecipitation was performed overnight with α-VP40 antibody in TNE150 + 0.1% NP-40 buffer. Protein A/G was added for 2 h and complexes were washed twice with TNE150 + 0.1% NP-40 and once with kinase buffer. Radioactive immunoprecipitated complexes were resuspended in Laemmli buffer, run on a 4–20% Tris-glycine SDS gel, dried, and exposed to phosphoroImager cassette. Densitometry analysis of kinase activity as determined by ImageJ software is shown as a percentage in the bottom panel, with untreated VP40-transfected 293T cells set to 100%. **(E)** Five hundred micrograms of CEM whole cell extract was immunoprecipitated with 10 μg of α-Cyclin E or normal rabbit IgG antibody in TNE50 + 0.1% NP-40, and incubated overnight at 4°C. Fifty microliters of a 30% slurry of Protein A/G was added next day, incubated for 2 h, washed twice with PBS and once with kinase buffer, and then resuspended in kinase buffer. Fifty microgram of 10–12 mer peptides matching potential phosphorylation sites on EBOV VP40 were added to 15 μL of sample IP and 2 μL of a [γ-^32^P] ATP and kinase buffer solution (1:3). Samples were incubated for 1 h before being dotted onto Whatman glass microfibre filters and dried for 30 min before being submerged in 1x TE buffer with gentle agitation for 2 days. DPM2 counts were then taken with a scintillation counter (QuantaSmart^TM^). The peptide sequences used are illustrated in the boxes. Underlined letters indicate the potentially phosphorylated residue. Residues that were altered from the wild type sequence are indicated (red).

Our previous results in **Figure 3A** indicated that the VP40 incorporated into exosomes was of a slightly higher molecular weight, suggesting a selective post-translational modification. It has previously been shown that VP40 is phosphorylated at tyrosine 13 (Y^13^) by c-Abl1 kinase, which was necessary for the productive replication of high titer EBOV *in vitro* (García et al., 2012). Therefore, we asked whether VP40 could potentially be phosphorylated in cells prior to packaging and release with exosomes. The VP40 amino acid sequence contains multiple potential sites of phosphorylation. Using our mass spectrometry data of EBOV virions we found Ser-233, Thr-272, Thr-277, and Ser-278 to be potentially phosphorylated (**Figures 4A,B**). The whole phosphoproteomic analysis of EBOV is ongoing and will be published elsewhere (data not shown). The whole phosphoproteomic analysis of EBOV is ongoing and will be published elsewhere (data not shown). Interestingly, Ser-233 of VP40 is part of a ^233^SPEKI^237^ sequence (shown in red; **Figure 4A**) which resembles the Cyclin-dependent kinase 2 (Cdk2) phosphorylation motif (S/T)PX(K/R) (Deng et al., 2002). To determine if Cdk2 is indeed involved in the phosphorylation of VP40, we designed *in vitro* kinase assays using cell extracts from which Cdk2 was immunoprecipitated with α-Cdk2 antibody. In addition, antibodies against Cyclin A and Cyclin E (α-CycA and α-CycE), which are normal partners of Cdk2, were utilized to precipitate specific Cdk2 kinase complexes. *In vitro* kinase assays were then performed using purified VP40 protein and [γ-^32^P] ATP. Results in **Figure 4C** indicate that there is a dramatic increase in VP40 phosphorylation in the presence of Cdk2 as compared to controls (lanes 1-3). VP40 phosphorylation additionally increased when the kinase was precipitated with either Cyclin E- or Cyclin A-specific antibodies (**Figure 4C**, lanes 4 and 5). This is consistent with the association of the Cyclins with Cdk2 in an active kinase complex, rather than Cdk2 alone which may represent a mixture of inactive complex (Cdk2 alone) with active complexes (Cdk2/CycE, Cdk2/CycA) in cells.

As further confirmation of the involvement of Cdk2, we then asked whether the phosphorylation of VP40 *in vitro* could be prevented with specific Cdk inhibitors. Cdk2 inhibitors with high selectivity such as Alsterpaullone, Indirubin-3′-monoxime, Purvalanol A, and r-Roscovitine have previously been shown to inhibit the replication of other viruses including HIV-1, Hepatitis C Virus, Human Cytomegalovirus, Adenovirus, and Varicella-Zoster Virus (Bresnahan et al., 1997; Taylor et al., 2004; Guendel et al., 2010; Cheng et al., 2013; Munakata et al., 2014; Roy et al., 2015). We therefore investigated if any of these inhibitors could affect the phosphorylation of VP40 *in vitro*. While Alsterpaullone and Indirubin-3′-monoxime resulted in only a moderate down-regulation of VP40 phosphorylation (**Figure 4C**, lanes 6 and 7), Purvalanol A was slightly more effective (**Figure 4C**, lane 8), and importantly, r-Roscovitine completely inhibited the phosphorylation of VP40 (**Figure 4C**, lane 9). Collectively, these data indicate that Cdk2/Cyclin E and Cyclin A complexes (markers of late G1 and early S phases of cell cycle) efficiently phosphorylate VP40 *in vitro*, and this phosphorylation can be prevented with specific Cdk inhibitors.

Cdk2/Cyclin E complexes are most active during the G1/S phase of the cell cycle, however, most cell lines used *in vitro* (i.e., 293T cells) are a mixed population of cells at the G1 (∼65%), S (∼20%), and G2/M (∼10%) phases. To obtain a more uniform population of cells at G1/S, we treated cells with Hydroxyurea (Hu), which is a reversible compound that targets early S phase DNA synthesis (Kashanchi et al., 2000). We transfected 293T cells with VP40 plasmid and added Hygromycin B for plasmid selection. Cells were either untreated or blocked at the G1/S phase with Hydroxyurea (2 mM) for 1 day. Cells were subsequently pulsed with radio-labeled [γ-^32^P] ATP and treated with r-Roscovitine for 4 h in complete media, followed by washes and immunoprecipitations using α-VP40 antibody. The data in **Figure 4D** show that VP40 is indeed phosphorylated *in vivo* and its phosphorylation is inhibited by r-Roscovitine treatment. Kinase activity as a percentage is quantified using densitometry as illustrated in the panels beneath **Figures 4C,D**.

In order to confirm the specific site of phosphorylation on VP40, we utilized five 10–12 mer specific peptides that span various residues that are potentially modified (as shown in **Figures 4A,B**). The expected target of Cdk2 phosphorylation was Ser 233, which is part of a SPEKI sequence that resembles Cdk2’s (S/T)PX(K/R) consensus phosphorylation site (Deng et al., 2002). The ADLTSPEKIQAI peptide (S3) and the S233A mutated ADLTAPEKIQAI peptide (S3M) were used in *in vitro* kinase assays. The Cyclin E-precipitated Cdk2 kinase complex was incubated with the peptide substrates and phosphorylation was detected using Whatman glass microfibre filters. Other potential targets were also examined using additional synthesized peptides containing Thr-277 (GKKVTAKNGQPI, T7), Ser-278 (GKKVASKNGQPI, S8), and Thr-272 (LVHKLTGKKV, T2) residues (**Figure 4E**). To distinguish between Thr-277 and Ser-278 residues, alanine mutations were introduced: S278A for T7 and T277A for S8. The S3 peptide containing the putative Cdk2 phosphorylation site showed the greatest level of phosphorylation by Cdk2/Cyclin E, which was abrogated in the S3M mutant (**Figure 4E**). Peptide T7 containing the Thr-277 site showed considerably reduced phosphorylation, and the peptides containing Thr-272 (T2) and Ser-278 (S8) showed only background levels of phosphorylation, similar to S3M peptide (**Figure 4E**). IP experiments were conducted using CEM extracts as we have previously shown that these cell extracts contain active Cdk/Cyclin kinases that can easily be purified with specific antibodies (Wang et al., 2001). Taken together, these results suggest that VP40’s Ser-233 is the target site for phosphorylation by Cdk2/Cyclin E.

### Presence of Ebola VP40 Results in Regulation of microRNA Machinery in Donor and Recipient Cells

**FIGURE 5:**
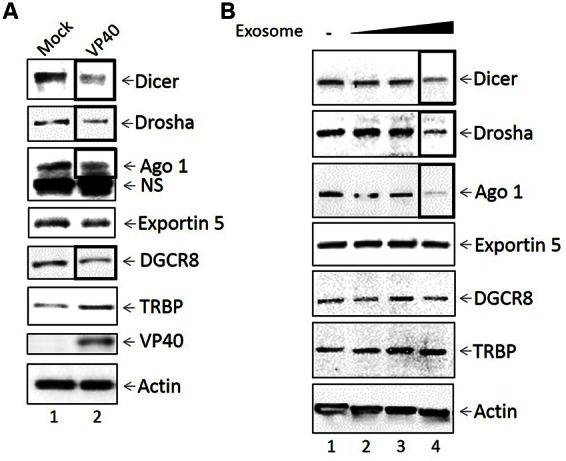
**Inhibition of miRNA machinery in transfected donor and recipient cells. (A)** Mid-log phase 293T cells were transfected with VP40-producing plasmids via electroporation and incubated for 24 h, followed by treatment with Hygromycin B for plasmid selection until cells were confluent (9 days; lane 2). Mock transfected cells were electroporated without plasmid (lane 1). Cells were lysed with lysis buffer, whole cell extract was resuspended in Laemmli buffer, run on a 4–20% Tris-glycine SDS gel, and subsequently analyzed by Western blot for levels of miRNA machinery components Dicer, Drosha, Ago 1, Exportin 5, DGCR8, and TRBP. Presence of VP40 protein was analyzed as a control, along with Actin. NS, non-Specific binding **(B)** VP40-transfected 293T cells were grown under antibiotic selection for 14 days, followed by removal and filtering (0.22 micron) of the supernatant. CEM cells were treated with 100, 250, or 500 μL of filtered transfection supernatant (0.3, 0.75, and 1.5 mU AChE, respectively) and incubated for 5 days at 37°C. Cell pellets were isolated, lysed with lysis buffer, and analyzed by Western blot for miRNA machinery components Dicer, Drosha, Ago 1, Exportin 5, DCGR8, and TRBP. Actin levels were also analyzed.

It has previously been shown that a downregulation of various microRNA machinery components such as Dicer, Drosha, Exportin 5 and Ago proteins have been linked to the induction of apoptosis (Su et al., 2009; Han et al., 2013; Bian et al., 2014; Lombard et al., 2015), while upregulation of Ago 1 has been linked to a slowing down of the cell cycle and eventual apoptosis (Su et al., 2009; Parisi et al., 2011: 1). Given that we observed apoptosis in cells treated with purified VP40 protein and supernatants from cells transfected with VP40 DNA, we next asked whether Ebola VP40 was able to affect the cellular miRNA machinery of transfected cells, as well as cells receiving exosome-laden transfected cell supernatant treatments. It has been previously shown that some viruses interfere with host miRNA machinery as a means of evading host immune responses important for cell signaling and regulation of apoptosis (Coley et al., 2010; Van Duyne et al., 2012). Additionally, it has been observed that EBOV proteins VP35, VP30, and potentially VP40 are capable of deregulating the host RNAi pathway as suppressors of RNA silencing (Fabozzi et al., 2011). Here, 293T cells were transfected with plasmids producing VP40 and kept under Hygromycin B antibiotic selection. Cell pellets were harvested, lysed, and analyzed by Western blot for levels of Dicer, Drosha, Ago 1, Exportin 5, DGCR8, TRBP, VP40 and Actin. Results in **Figure 5A** show a down-regulation of Dicer, Drosha, Ago 1, and DGCR8 protein levels in cells transfected with VP40 DNA in comparison to mock transfected cells. However, the levels of Exportin 5 remained constant while TRBP levels slightly increased.

We next examined the effects of exosomes from transfected cells on recipient cell miRNA machinery. Exosomes were purified from VP40-transfected cells using a 0.22 micron filter and ultracentrifugation, followed by treatment of recipient CEM cells with varying concentrations of exosomes (0.3, 0.75, and 1.5 mU AChE). Recipient cells were treated for 5 days, harvested, and analyzed by Western blot for the components of the miRNA machinery, along with accessory proteins including TRBP and DGCR8. Interestingly, a similar decrease was observed in the expression of Dicer, Drosha and Ago 1 in lymphocytes treated with the highest concentration of exosomes (**Figure 5B**). TRBP, DGCR8, and Exportin 5, on the other hand, were unaffected by VP40-associated exosomes. Together, these data suggest that VP40 may be capable of dysregulating some specific components of miRNA machinery components not only in infected donor cells, but also in naïve neighboring cells receiving VP40-containing exosomes.

### Treatment of VP40-Transfected 293T Cells with Oxytetracycline Decreases Exosomal Release and Reduces Recipient Cell Dysregulation

**FIGURE 6:**
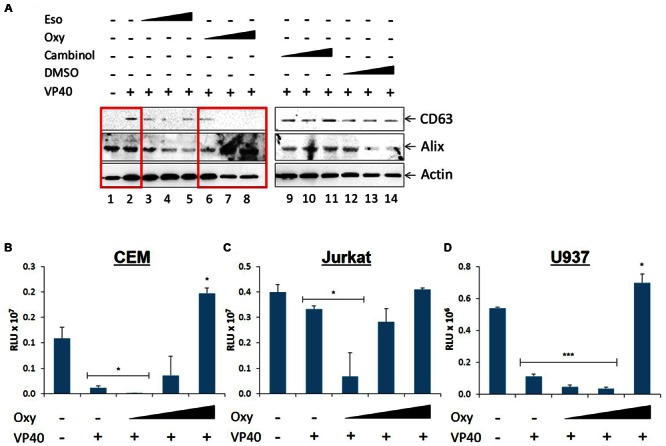
**Effect of FDA approved drugs on EBOV transfected donor and recipient immune cells. (A)** Varying concentrations of Cambinol and Esomepraole (1 nM, 1 μM, 10 μM), Oxytetracycline (1 nM, 10 nM, 10 μM), and DMSO (0.00001, 0.0001, 0.001%) treatments were administered to VP40-transfected 293T cells, followed by incubation for 3 days. Supernatant was centrifuged to remove cellular material and subsequently incubated for 72 h at 4°C with 20 μL of 30% slurry of NT80/82 beads. The NT pellet was washed twice with PBS and resuspended in SDS Laemmli buffer, followed by Western blot analysis for levels of exosomal markers CD63 and Alix. Actin levels were also analyzed. Mid-log phase EVTR2C cells (containing VP40) were treated with either low (0.1 μM), medium (1 μM), or high (10 μM) concentrations of Oxytetracycline and incubated for 5 days along with untreated 293T cells. Cell-free supernatants were harvested, filtered, and used to treat CEM **(B)**, Jurkat **(C)**, and U937 **(D)** cells. Cells were incubated for 5 days and assayed with CellTiter-Glo for viability. Statistical significance was determined using two-tailed student’s *t*-test with all groups compared to the untreated 293T cell control groups. RLU, Relative Luminescent Units. ^∗^*p* < 0.05, ^∗∗∗^*p* < 0.001.

We next asked whether treatment of VP40-transfected 293T cells with FDA-approved drugs would be able to down-regulate the release of exosomes, and thereby potentially mitigate the detrimental effects seen in recipient cells. We screened a panel of FDA- and non-FDA-approved drugs to find that Esomeprazole, Oxytetracycline, and Cambinol reduce exosomal release in HIV-1 infected cells (unpublished data). Here, 293T cells were transfected with VP40 DNA, placed under antibiotic selection, and treated with increasing concentrations of 1 nM to 10 μM of Esomeprazole, Oxytetracyline, Cambinol, or DMSO as control. Cells were then pelleted, lysed, and analyzed by Western blot for classic exosomal markers CD63 and Alix, as well as Actin. Results in **Figure 6A** show an increase in CD63 and Actin in cells transfected with VP40 DNA in comparison to untransfected controls (lanes 1 and 2). In addition, a decrease in CD63 and Actin was observed in transfected cells treated with increasing concentrations of Oxytetracycline (**Figure 6A**, lanes 6–8). Treatment of cells with increasing concentrations of Esomeprazol, Cambinol, and DMSO resulted in no significant differences in CD63, Alix, or Actin levels.

As Oxytetracycline showed a positive effect of potentially down-regulating exosomal release from donor cells, we then asked whether this same treatment on donor cells would reduce the inhibitory effects we observed on recipient immune cells. We placed our VP40-resistant EVTR2C cells under varying concentrations (100 nm-10 μM) of Oxytetracycline and incubated for 5 days. Supernatants were harvested, filtered through a 0.22 micron filter, and then used to treat CEM, Jurkat, or U937 recipient cells. Treatment of immune cells with untreated, filtered 293T cell supernatant served as a negative control. Cell viability was then assayed with CellTiter-Glo. Results in **Figures 6B,D** show an expected decrease in cell viability in the three cell types treated with EVTR2C supernatant. Interestingly, CEM and Jurkat cells treated with 0.1, 1.0, and 10 μM Oxytetracycline showed a corresponding increase in cell viability in a dose-dependent manner (**Figures 6B,C**). U937 cells showed no significant change in cell viability until treatment with the highest dose of Oxytetracycline (**Figure 6D**). Collectively, these data indicate that an increase in exosomal release from cells may occur in the presence of VP40. Additionally, treatment with Oxytetracycline may cause a reduction of exosomal release in VP40-producing cells, and thereby diminish the inhibitory effects on neighboring recipient immune cells.

### Effect of Ebola VP40 Presence on Exosomal Markers and ESCRT Machinery

**FIGURE 7:**
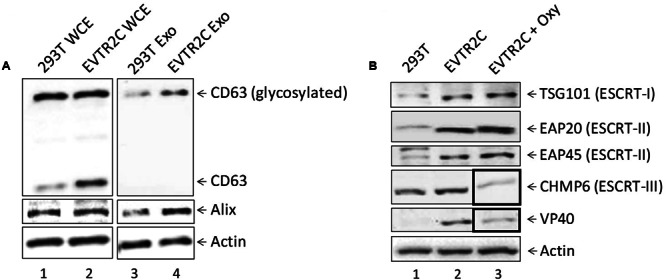
**Effect of VP40 presence on exosomal markers and ESCRT machinery components. (A)** 293T cells and VP40-resistant clones (EVTR2C cells) were grown for 5 days, followed by harvesting of both cells and supernatants. Cells were lysed and analyzed by Western blot for levels of CD63, Alix, and Actin. Supernatants were centrifuged and passed through a 0.22 micron filter, followed by incubation at 4°C with 30% slurry of NT80/82 beads for 72 h. Beads were spun down next day and resuspended in Laemmli buffer, followed by SDS/PAGE and Western blot analysis for levels of CD63, Alix, and Actin. **(B)** Whole cell extracts from 293T, EVTR2C, and EVTR2C cells treated for 5 days with 10 uM Oxytetracycline were run on a 4–20% Tris-glycine SDS gel and analyzed by Western blot for levels of ESCRT pathway components TSG101 (ESCRT-I), EAP20 (ESCRT-II), EAP45 (ESCRT-II), and CHMP6 (ESCRT-III), as well as VP40 and Actin.

We have previously shown that HIV-1-infected cells have increased levels of cytosolic unprocessed CD63, while exosomes from HIV-1-infected cells contain increased levels of glycosylated CD63, indicating that infection results in increased CD63 production or exosome biogenesis (Narayanan et al., 2013; Sampey et al., 2016). We likewise observed a similar increase in the levels of CD63 in exosomes in presence of VP40 (**Figure 6A**, lane 2). To further characterize this change, we used NT80/82 beads to concentrate the exosomes in supernatants from 293T and EVTR2C cells that were simultaneously harvested and lysed. Both exosomes and cellular extracts were analyzed by Western blot for levels of CD63, Alix, and Actin. Results in **Figure 7A** (left panel) show an increase in CD63 levels in the presence of VP40 compared to control cells. However, the intracellular levels of glycosylated CD63 and Alix remained similar. In contrast, Alix and glycosylated CD63 increased in exosomes from EVTR2C cells in comparison to controls (**Figure 7A**, right panel). These data indicate that VP40 may induce an increased total level of CD63 or an increased production of exosomes, similar to the HIV-1-infected cell phenotype.

As exosomal biogenesis and release is governed largely by the ESCRT pathway, we next asked if components of the ESCRT machinery were being affected by the presence of VP40 in our resistant clones. EVTR2C and 293T cells were cultured to mid-log phase, harvested, and lysed. Whole cell extracts were analyzed by Western blot for levels of ESCRT I (TSG101), II (EAP20, EAP45), and III (CHMP6) proteins, as well as VP40. The data in **Figure 7B** show an increase in levels of TSG101, EAP20, and EAP45 proteins in EVTR2C cells in comparison to 293T cells. Western blot also confirmed the presence of VP40 in EVTR2C cells. Finally, as we had observed a decrease in exosomal release with Oxytetracycline treatment (**Figure 6A**, lanes 6–8), we hypothesized that ESCRT components may be dysregulated by this drug treatment. We treated EVTR2C cells with Oxytetracycline for 5 days followed by lysis and Western blot for ESCRT I–III protein components, as well as VP40 protein and Actin. Data in **Figure 7B** (lane 3) shows that treatment with Oxytetracycline resulted in a downregulation of ESCRT III protein CHMP6. Surprisingly, levels of intracellular VP40 were also decreased in EVTR2C cells treated with Oxytetracycline. Together, these results indicate that exosomal markers such as CD63 and specific ESCRT machinery components may be upregulated in VP40-producing cells, correlating with a possible increase in exosome production. Treatment of VP40-containing cells with Oxytetracycline may also offer a potential tool for decreasing the output of exosomes through inhibition of a CHMP6-mediated pathway, or by inhibiting the production or accumulation of VP40 within cells.

## Discussion

Ebola virus can cause severe hemorrhagic fever in humans and non-human primates, resulting in up to 80–90% mortality rates and long-term morbidity in convalescent survivors (Elshabrawy et al., 2015; Martines et al., 2015; Singh et al., 2015). Infection is characterized by prolific production of cytokines, unregulated viral replication in migrating immune cells of the myeloid lineage (dendritic cells, monocytes/macrophages), Kupffer cells in the liver, endothelial and epithelial cells, as well as widespread bystander lymphocyte apoptosis (Geisbert et al., 2003). In fatal cases, the adaptive immune (T- and B-cell-mediated) response is strongly repressed due to extensive cell death, despite a lack of productive infection in these cells, however, survivors of EBOV infection are able to produce the appropriate adaptive immune responses in the absence of bystander lymphocyte apoptosis (Geisbert et al., 2000, 2003). Our data using various forms of VP40-containing complexes showed a dysregulation of treated immune cells. We observed that VP40, when enclosed by membranes and masked by GP within VLPs, showed minimal toxicity toward both T-cells and monocytes. However, when treated with free VP40, we observed cell death in T-cells through the induction of Caspase 3 and cleavage of PARP-1. We next analyzed the effects of Ebola protein-transfected cell supernatants on T-cells and monocytes. VP40-transfected cell supernatants, both filtered (0.22 micron) and unfiltered, resulted in consistent amounts of cell death amongst CEM, Jurkat, and U937 cells, as well as U937 MDMs and 3 primary MDMs, although there was some level of cell death from NP- and GP-transfected cell supernatants in non-differentiated recipient cells. Furthermore, treatment with supernatants from VP40-resistant clones (selected under Hygromycin B), resulted in cell death in the primary MDMs, similar to treatment with VP40-transfected cell supernatants. Interestingly, cell death occurred in monocytes when treated with supernatants from VP40-transfected cells, but not when treated with *E. coli*-purified free VP40, suggesting that VP40 produced in eukaryotic cells may have a different phenotype. We have previously shown that exosomes are able to be captured and concentrated with the use of specific nanotrap particles (NT80/82) (Narayanan et al., 2013; Jaworski et al., 2014a,b; Sampey et al., 2016). Similarly, we were able to capture exosomes from VP40-transfected cell supernatants with NT80/82 beads. Furthermore, Western blot of these NT samples showed that VP40 is indeed packaged into exosomes. These data from EBOV VP40 is similar to our recent study of exosomes from cells infected with Rift Valley Fever Virus (enveloped ssRNA virus), that were also found to regulate neighboring, uninfected immune cells by destroying T-cells and monocytes (Ahsan et al., 2016). This could potentially represent a new pathogenesis paradigm for ssRNA viruses. Exosomes from EBOV-infected cells may then also contain Ebola viral proteins including, but perhaps not limited to, VP40 or other viral transcripts. Here, we show that VP40 incorporated into exosomes is capable of inducing cell death in uninfected T-cell and monocyte populations, a phenotype that is strongly reminiscent of bystander lymphocyte apoptosis.

Currently, most Ebola research is restricted to work within a BSL-4 biocontainment facility, greatly limiting diagnosis, sample analysis, and *in vivo* studies. Therefore, we wished to determine if it were possible to use nanotrap particles under strongly reducing buffer conditions to safely and effectively detect viral proteins in human samples. We were able to ascertain an effective nanoparticle (NT219; Cibacron Blue F3G-A bait with VSA shell) capable of binding to and concentrating Ebola VP40 protein within VLPss spiked into human saliva or urine in the presence of stringent SDS buffer (1:1; RIPA and Laemmli buffer). The binding efficiency of our NT219 under these conditions was approximately ∼1%; however, the sensitivity of this assay could be greatly increased with the use ELISA rather than Western blot for detection. Improvement of this method could be a significant enhancement to our current diagnostic methods, allowing detection of Ebola proteins in human samples to safely be carried out in simple BSL-1 or -2 conditions or in the field with minimal PPE and biocontainment requirements.

We observed that the VP40 found within exosomes migrated slower on SDS/PAGE in comparison to that found within VLPs. It has previously been shown that VP40 is phosphorylated at tyrosine 13 (Y^13^) by c-Abl1 (García et al., 2012), which is required for high levels of replication of EBOV *in vitro*. In this study, we wished to see if VP40 was similarly modified prior to packaging and release into exosomes. We found that VP40 is in fact phosphorylated by Cdk2 in complex with either Cyclin A or Cyclin E, although Cdk2/CycE appears to be a stronger kinase for VP40 at the G1/early S phase. Additionally, we were able to prevent the kinase activity of Cdk2 on VP40 with specific inhibitor r-Roscovitine treatment. To determine the site of phosphorylation, we designed five 10-12 mer peptides matching VP40 residues for subsequent *in vitro* kinase assay. Using this method, we found that Ebola VP40 is mostly phosphorylated by Cdk2/CycE on Ser-233, representing a potential drug target.

Previous studies have shown that exosomes from virally infected cells can contain functional miRNAs that are capable of dysregulating naïve recipient cells (Pegtel et al., 2010, 2011; Barth et al., 2011; Sampey et al., 2016). Thus, we wished to determine if the apoptosis we observed in immune cells treated with exosomes containing VP40 was due to the regulation of miRNA in recipient cells. Here, we show that VP40-transfected cells have lower levels of Dicer, Drosha, Ago 1, and DGCR8 compared to controls. Additionally, we show for the first time that exosomes from Ebola VP40-transfected cells are capable of downregulating the levels of Dicer, Drosha, and Ago 1 in naïve recipient T-cells. Infection with flaviviruses, including Dengue virus and Kunjin virus, has been previously shown to result in the inhibition of RNAi machinery and a downregulation of Drosha and DGCR8 (Moon et al., 2015; Casseb et al., 2016). We also have previously shown that the HTLV-1 protein Tax can directly modulate the RNAi pathway through the shuttling of Drosha and Dicer to the proteasome for degradation with no induction of apoptosis (Van Duyne et al., 2012). Alternatively, the downregulation of various miRNA machinery components including Dicer, Drosha, Exportin 5 and Ago proteins have, in many cases, been linked to the induction of apoptosis (Su et al., 2009; Han et al., 2013; Bian et al., 2014; Lombard et al., 2015). In combination, these data support the suggestion that EBOV VP40 within cells and incorporated into exosomes can modulate RNAi components in both parent donor and recipient immune cells, ultimately resulting in cell death. This may therefore represent a novel mechanism of immune system evasion by EBOV by inducing bystander lymphocyte apoptosis in uninfected recipient T-cells through the delivery of VP40-laden exosomes from infected donor cells, during the course of infection.

In this manuscript we found that levels of the exosomal marker CD63 increase in cells producing EBOV VP40. A side-by-side comparison of EVTR2C cellular lysates to EVTR2C exosome samples (obtained with NT80/82 beads) showed an increase in CD63 levels within cells and a corresponding increase in glycosylated CD63 and Alix in exosomal samples, potentially correlating to an increase in exosomal biogenesis and release. These data are supported by previous observations of increasing CD63 levels in exosomes and from cells infected with HIV-1 and HTLV-1 (Narayanan et al., 2013; Jaworski et al., 2014a; Sampey et al., 2016). Recently, we have found several FDA- and non-FDA-approved drugs that have been able to reduce exosomal release in HIV-1-infected cells (unpublished data). Along these lines, we wished to see if any of these drugs (Esomeprazol, Oxytetracycline, and Cambinol) would be effective in reversing the implied increase in exosome biogenesis seen in cells transfected with EBOV VP40. Our initial results indicated that CD63 levels decreased in exosomes with increasing concentrations of Oxytetracycline treatment in a dose-dependent manner. Additionally, a recovery of cell viability in recipient naïve immune cells was achieved with supernatant from Oxytetracycline treated VP40-resistant clones.

The ESCRT pathway, composed of ESCRTs -0, -I, -II, -III complexes, and ATPase VPS4, is the main pathway responsible for the sorting of cargo into and release of exosomes from host cells. Early ESCRT complexes are responsible for recognizing and sorting cargo, as well as recruiting later ESCRT complexes, which in turn are responsible for membrane budding, scission, and release of vesicles. VPS4 is responsible for final membrane fusion and recycling of ESCRT complex components (Henne et al., 2011). While the ESCRT pathway is important for exosomal release and other cellular processes, recent studies have found evidence that a wide assortment of enveloped viruses (i.e., HIV-1, SIV, HTLV-1, Lassa Fever Virus, HBV, Rabies Virus, Japanese Encephalitis Virus, etc.) are able to hijack and utilize ESCRT complexes in order to bud from the plasma membrane (Votteler and Sundquist, 2013). Indeed, Ebola VP40 is capable of recruiting TSG101 (ESCRT-I) and NEDD4 family proteins via two different domains as a means of utilizing the ESCRT pathway for viral budding (Harty et al., 2000; Martin-Serrano et al., 2001; Timmins et al., 2001, 2003; Licata et al., 2003; Yasuda et al., 2003). Therefore, to further characterize the mechanism of action of exosomal release in the presence of EBOV VP40, we analyzed VP40-resistant clones either alone or treated with Oxytetracycline for levels of ESCRT complex proteins. We found that the levels of TSG101 (ESCRT-I), as well as EAP20 and EAP45 (ESCRT-II) increased in the presence of VP40. This resembles a pattern seen in HIV-1 infection, where Nef and Gag regulate the release of exosomes through the manipulation and recruitment of ESCRT-I and ESCRT-III complexes (Lenassi et al., 2010). In addition, we show that with Oxytetracycline treatment the levels of CHMP6 (ESCRT-III) decrease in the VP40-resistant clones, which may represent a method by which exosomal biogenesis and release may be inhibited. Surprisingly, we also observed that the levels of VP40 inside EVTR2C cells decreased in the presence of Oxytetracycline. Future experiments will further characterize this phenomenon and determine the mechanism by which Oxytetracycline or other Tetracycline derivatives may affect intracellular or extracellular levels of VP40 in EVTR2C cells. Collectively, these data may present a potential novel therapeutic option for the treatment of EBOV infection with an FDA-approved antibiotic to prevent progressing pathogenesis and bystander apoptosis.

While Ebola has not been considered as a virus exhibiting latency, the proposed reservoir of Ebola (i.e., bats) can possess one or more functionally maintained copies of filovirus-like genes within their genomes, indicating that despite the virus’ cytoplasmic replication and lack of viral reverse transcriptase, successful integration into the mammalian host genome is possible (Belyi et al., 2010; Taylor et al., 2010, 2011). Specifically, the nucleocapsid (NP), matrix (VP40), and potentially GP genes from Ebola have been discovered to be integrated into the bat genome (Belyi et al., 2010). Therefore, cases of viral recurrence and persistence in survivors of infection could potentially be explained by a true viral latency from an integrated form of EBOV (potentially through cellular telomerase enzyme which contains RT activity). Numerous studies have demonstrated that cellular proteins, mRNAs and miRNAs from many different cell types can be sorted and released in exosomes to affect neighboring cells (Lotvall and Valadi, 2007; Skog et al., 2008). Furthermore, previous studies have described the packaging of viral proteins and miRNAs into exosomes, as well as the transfer of these exosomes into uninfected, neighboring cells to alter cellular activity (Pegtel et al., 2010, 2011; Barth et al., 2011; Pegtel et al., 2011; Narayanan et al., 2013; Ahsan et al., 2016; Sampey et al., 2016). It therefore stands to reason that if a form of EBOV may potentially be integrated into the human host genome, that any products of this proviral form might be passed to other healthy cells through exosomes, and thereby induce some recurrence of illness. Along these lines, positive PCR results in cases of EBOV disease recurrence may not be from entire, replicative virus, but possibly from pieces of the EBOV genome that have integrated into the host genome.

**FIGURE 8:**
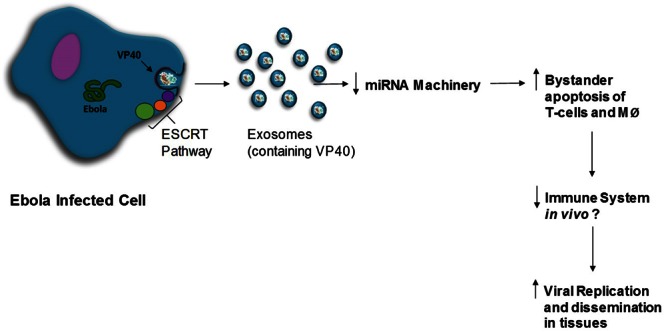
**Model for Ebola VP40 role in pathogenesis.** VP40 within the Ebola-infected host cell will interact with the ESCRT pathway and be packaged into exosomes. Exosomes containing EBOV VP40 exit the cell and downregulate miRNA machinery (i.e., Dicer, Drosha, Ago 1) in recipient cells, including T-cells and monocytes/macrophages (MØ). Subsequent bystander lymphocyte apoptosis is increased, potentially resulting in immune system repression and increased viral replication in various compartments of the host.

In this study, we sought to discover if Ebola structural proteins (NP, GP, and VP40) can be packaged into exosomes to affect other neighboring cells. It has been demonstrated previously that direct infection of cells with EBOV does not result in the apoptosis of the infected cells. Rather, it has been shown that infected cells take part in a non-apoptotic form of cell death: necrosis (Olejnik et al., 2013; Falasca et al., 2015). In contrast, the cell death by apoptosis of uninfected, bystander lymphocytes has been observed under many different circumstances (Baize et al., 1999, 2000, 2002; Geisbert et al., 2000, 2003; Bradfute et al., 2007, 2010; Gupta et al., 2007; Wauquier et al., 2010), although the molecular mechanism for the induction of apoptosis has not been defined. Here, we have shown for the first time that Ebola VP40 is able to be packaged into host cell exosomes, and that these exosomes can negatively impact the viability of recipient naïve immune cells, potentially causing bystander lymphocyte apoptosis in T-cell populations (**Figure 8**). We therefore speculate that in an *in vivo* infection there is a significant contribution of EBOV protein-containing exosomes to pathogenesis and poor prognosis. As such, we have provided a possible method of prevention of this exosomal packaging and release with the use of FDA-approved antibiotics such as Oxytetracycline, which could allow for the development of an effective adaptive immune response in infected patients and providing a better chance of survival.

## Author Contributions

MP and AM contributed equally to generation of data and writing of the manuscript. CD and AS contributed to some manuscript writing and experimental procedures, especially **Figure 7**. YA and RB contributed to ultracentrifugation and isolation of exosomes, as well as AchE assays. SI and GS contributed to plasmid design and purification, transfections and selection of clones. BL contributed nanoparticles. SN performed proteomic analyses of EBOV VP40 for **Figure 4** in conjunction with PI and AB. MA provided various reagents and FK contributed to the overall direction and coordination of the project, as well as experimental design, quantitative analyses, and data interpretation.

## Conflict of Interest Statement

The authors declare that the research was conducted in the absence of any commercial or financial relationships that could be construed as a potential conflict of interest.
