# Corrigendum: Ebola VP40 in Exosomes Can Cause Immune Cell Dysfunction

**DOI:** 10.3389/fmicb.2018.00692

**Published:** 2018-04-17

**Authors:** Michelle L. Pleet, Allison Mathiesen, Catherine DeMarino, Yao A. Akpamagbo, Robert A. Barclay, Angela Schwab, Sergey Iordanskiy, Gavin C. Sampey, Benjamin Lepene, Philipp A. Ilinykh, Alexander Bukreyev, Sergei Nekhai, M. Javad Aman, Fatah Kashanchi

**Affiliations:** ^1^Laboratory of Molecular Virology, School of Systems Biology, George Mason University, Manassas, VA, USA; ^2^Department of Physiological Sciences, Eastern Virginia Medical School, Norfolk, VA, USA; ^3^Department of Microbiology, Immunology and Tropical Medicine, Research Center for Neglected Diseases of Poverty, George Washington University School of Medicine and Health Sciences, Washington, DC, USA; ^4^Department of Medicine, University of North Carolina HIV Cure Center, University of North Carolina at Chapel Hill School of Medicine, Chapel Hill, NC, USA; ^5^Ceres Nanosciences Inc., Manassas, VA, USA; ^6^Departments of Pathology, University of Texas Medical Branch at Galveston, Galveston, TX, USA; ^7^Microbiology and Immunology, University of Texas Medical Branch at Galveston, Galveston, TX, USA; ^8^Galveston National Laboratory, University of Texas Medical Branch at Galveston, Galveston, TX, USA; ^9^Department of Medicine, Center for Sickle Cell Disease, Howard University, Washington, DC, USA; ^10^Integrated BioTherapeutics, Inc., Gaithersburg, MD, USA

**Keywords:** Ebola virus, EBOV, VP40, VLP, exosomes, ESCRT, microRNA, apoptosis

In the original publication, Philipp A. Ilinykh and Alexander Bukreyev were not included as authors. The omission of these authors was a miscommunication among proteomics colleagues.

Secondly, there should be a change to Materials and Methods section. In the subsection “Identification of Potential Phosphorylation Sites in VP40 Protein,” in the first paragraph, the first sentence should read as follows: “Mass spectra for VP40 was obtained for this manuscript as previously described in our analysis of VP30 from EBOV virions (Ilinykh et al., 2014). Proteomics data for VP40 had not been previously published.”

For the third amendment, proteomics data on EBOV virions were mentioned as published whereas these data were not yet published. A correction should be made to Results section. In the sub-section “Ebola VP40 is Phosphorylated by Cyclin-Dependent Kinase 2,” in the first paragraph, the sentence should read as follows: “Using our mass spectrometry data of EBOV virions we found Ser-233, Thr-272, Thr-277, and Ser-278 to be potentially phosphorylated (Figures 4A,B). The whole phosphoproteomic analysis of EBOV is ongoing and will be published elsewhere (data not shown).”

The authors apologize and state that these corrections do not change the scientific conclusions of the article in any way.

The original article has been updated.

## Author contributions

MP and AM contributed equally to generation of data and writing of the manuscript. CD and AS contributed to some manuscript writing and experimental procedures, especially Figure 7. YA and RB contributed to ultracentrifugation and isolation of exosomes, as well as AchE assays. SI and GS contributed to plasmid design and purification, transfections and selection of clones. BL contributed nanoparticles. SN performed proteomic analyses of EBOV VP40 for Figure 4 in conjunction with PI and AB. MA provided various reagents and FK contributed to the overall direction and coordination of the project, as well as experimental design, quantitative analyses, and data interpretation.

### Conflict of interest statement

The authors declare that the research was conducted in the absence of any commercial or financial relationships that could be construed as a potential conflict of interest.
